# *Artemisia iwayomogi* plus* Curcuma longa* Synergistically Ameliorates Nonalcoholic Steatohepatitis in HepG2 Cells

**DOI:** 10.1155/2017/4390636

**Published:** 2017-10-17

**Authors:** Hyeong-Geug Kim, Sung-Bae Lee, Jin-Seok Lee, Won-Young Kim, Seung-Hoon Choi, Chang-Gue Son

**Affiliations:** ^1^Liver and Immunology Research Center, Daejeon Oriental Hospital of Oriental Medical College of Daejeon University, 176-9 Daeheung-ro, Jung-gu, Daejeon 301-724, Republic of Korea; ^2^Department of Medical Consilience, Dankook University, 152 Jukjeon-ro, Suji-gu, Yongin-si, Gyeonggi-do 16890, Republic of Korea

## Abstract

The combination of* Artemisia iwayomogi* and* Curcuma longa* radix is frequently prescribed for liver diseases in TKM. However, the synergic effects of the two herbs on nonalcoholic steatohepatitis (NASH) have not yet been studied. Therefore, we investigated the anti-NASH effects of the water extract of* A. iwayomogi* (AI),* C. longa *radix (CL), and combination of the two herbs (ACE). Hepatic steatosis and NASH were induced in HepG2 cells by treatment with palmitic acid (PA, for 6 h) with/without pretreatment of ACE (25 or 50 *μ*g/mL), AI (50 or 100 *μ*g/mL), CL (50 or 100 *μ*g/mL), curcumin (5 *μ*g/mL), or scopoletin (5 *μ*g/mL). The PA treatment (200 *μ*M) drastically altered intracellular triglyceride levels, total cholesterol, and expression levels of genes related to lipid metabolism (CD36, SREBP1c, PPAR-*γ*, and PPAR-*α*), whereas pretreatment with ACE significantly attenuated these alterations. ACE also protected HepG2 cells from PA- (300 *μ*M-) induced endoplasmic reticulum (ER) stress and apoptosis and attenuated the related key molecules including GRP78, eIF2, and CHOP, respectively. In conclusion, we found synergic effects of* A. iwayomogi* and* C. longa* on NASH, supporting the clinical potential for fatty liver disorders. In addition, modulation of ER stress-relative molecules would be involved in its underlying mechanism.

## 1. Introduction

Nonalcoholic fatty liver diseases (NAFLDs) include pathological conditions ranging from simple steatosis to nonalcoholic steatohepatitis (NASH). NAFLD is a major cause of chronic liver disorders, especially in developed countries, and the prevalence of NAFLD has been reported as 9% to 40% and 15% to 40% of the general populations of Asia and America, respectively [1]. Simple fat accumulation in the liver does not generally affect health; however, 10% of subjects with fatty liver progress to NASH, ultimately leading to liver cirrhosis and hepatocellular carcinoma [2, 3].

The details of NASH pathogenesis are not yet known, but the “multi-hit” theory is widely accepted [4]. With the consumption of a high-calorie and high-fat diet, obesity, hyperglycemia, hypertension, and diabetes, an increased flux of fatty acids into the liver results in hepatic steatosis. Severe hepatic steatosis induces multi-hit events, such as oxidative stress, lipid peroxidation, the influx of endotoxins from gastrointestinal tract, and mitochondrial dysfunction, which cause liver inflammation [5–7]. Therefore, fat accumulation and its related inflammatory responses are critical steps in NAFLD progression, and many studies have focused on these steps as therapeutic targets [8–10].

In traditional Korean medicine (TKM) theory, fat accumulation in the liver is defined as hepatic dysfunction by* “dampness and phlegm”* (濕痰) and* “blood stasis”* (瘀血) [11]. The herb* Artemisia iwayomogi* is frequently used to treat* “dampness and phlegm”* in TKM, and it also exerts effects against hyperlipidemia and obesity [12, 13].* Curcuma longa* radix, the therapeutic and antihyperlipidemic properties of which are well-documented, has been used to cure pathological* “blood stasis”* [14, 15]. Also, their major active compounds, scopoletin in* Artemisia iwayomogi* or curcumin in* Curcuma longa*, are also known partially or clinically to have the pharmaceutical actions on NASH or metabolic syndrome such as arteriosclerosis and hyperlipidemia [16–18].

The combination of* A. iwayomogi* and* C. longa* radix is frequently adapted in TKM clinical practices. However, the synergic effects of those two herbs on hepatic steatosis and NASH have not been studied until now. Herein, we evaluated the anti-NASH effects of a combination of* A. iwayomogi* and* C. longa* radix and investigated the corresponding mechanisms using an* in vitro* NAFLD and NASH model.

## 2. Materials and Methods

### 2.1. Preparation and Fingerprinting of ACE

*A. iwayomogi* and* C. longa* radix were purchased from Jeong-Seong traditional medicine company (Daejeon, South Korea). For extracts, 100 g samples of* A. iwayomogi* and* C. longa* radix were mixed separately in 1 L of 30% ethanol and shaken at 150 rpm overnight in a shaking incubator (Vision Scientific Co., Seoul, South Korea). The supernatant was centrifuged for 15 min at 150 ×g and filtered through filter paper (Dublin, CA, US). The filtered extract was lyophilized using a vacuum freeze-drying system and stored at −20°C. Finally, each 30% ethanol extract of* A. iwayomogi* (AI, the final yield of which was 10.22%) and* C. longa* (CL, the final yield of which was 4.89%) was obtained and stored at −20°C until use. An equal ratio of each extract (1.0 g of AI and 1.0 g of CL) was combined (ACE) for subsequent experiments. Before treatment with ACE, AI, and CL in HepG2 cells, these powders were dissolved in distilled water and filtered through a 0.45 *μ*m syringe filter.

### 2.2. Fingerprinting of ACE

**Figure 1:**
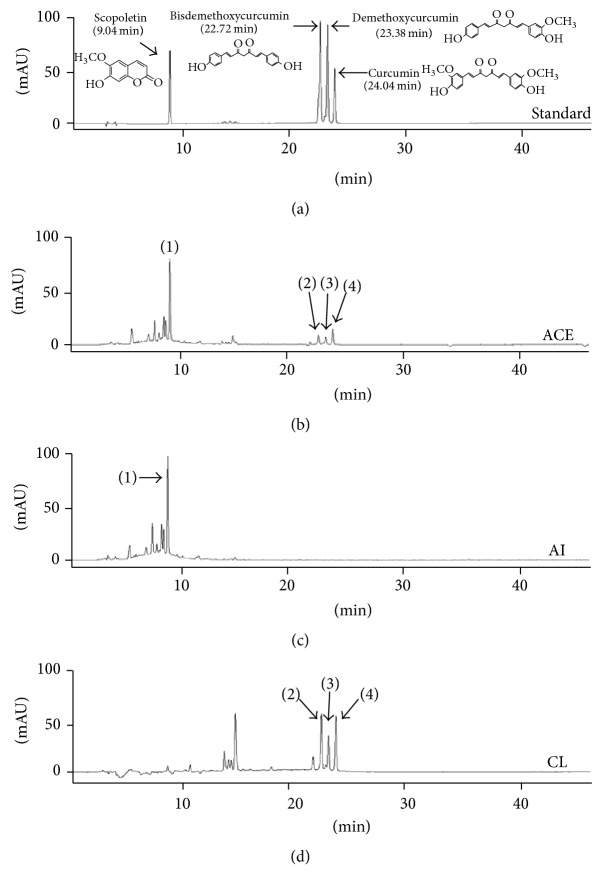
Fingerprint analysis of ACE using HPLC. (a) The four reference components, (b) ACE, (c) AI, and (d) CL were analyzed by HPLC. (1), (2), (3), and (4) are scopoletin, bisdemethoxycurcumin, demethoxycurcumin, and curcumin, respectively.

Fingerprinting analysis of ACE, AI, and CL was conducted using ultra-high-performance liquid chromatography-tandem mass spectrometry (UHPLC-MS/MS, Santa Clara, CA). A total of 5 mg of the ACE, AI, and CL samples was dissolved in 1 mL of 90% methanol, and the solution was filtered (0.45 *μ*m). From each sample solution, 10 *μ*L was subjected to UHPLC-MS/MS using an LTQ Orbitrap XL linear ion-trap MS Spectrometer (San Jose, CA). Separation was performed on an Accela UHPLC system using an Acquity BEH C18 column (1.7 *μ*m, 100 × 2.1 mm; Waters, Milford, Connecticut). The column was eluted at a flow rate of 0.4 mL/min using water (in 0.1% formic acid) and acetonitrile (in 0.1% formic acid), which were used as mobile phases A and B, respectively. The following gradients were applied: 0-1 min, 0-1% B in A; 1–7 min, 1–100% B in A; 7–10 min, 100-1% B in A (linear gradient). Compositional analyses were conducted using a photodiode array at 200–600 nm. Full-scan mass spectra were acquired at 150–1500 *m*/*z* in positive and negative modes. An Orbit rap analyzer was used for high-resolution mass data acquisition with a mass resolving power of 30,000 FWHM at 400 *m*/*z*. Tandem mass (MS/MS) spectra were acquired in data-dependent mode by collision-induced dissociation. For quantitative analysis, four reference compounds (scopoletin for* A. iwayomogi* and bisdemethoxycurcumin, demethoxycurcumin, and curcumin for* C. longa* radix) were used (Figure 1).

### 2.3. Cell Culture

The HepG2 cell line (murine hepatocellular carcinoma cells) was obtained from the Korean Cell Line Bank (Seoul, South Korea). The cells were cultured in Dulbecco's Modified Eagle's Medium supplemented with 10% fetal bovine serum (FBS) and antibiotics (100 U/mL penicillin G and 100 *μ*g/mL streptomycin). The cells were maintained under humidified conditions at 37°C in 5% CO_2_.

### 2.4. *In Vitro* Model of NAFLD and NASH and Drug Treatment

In vitro model of NAFLD and NASH referred to in previous study; the models were modified to clinically access [19]. Palmitic acid (PA) was dissolved in a 10% solution of bovine serum albumin (BSA) and shaken overnight at 37°C. Finally, an 8 mM stock solution of PA was obtained. Then the PA was used to treat HepG2 cells (200 *μ*M for the NAFLD model or 300 *μ*M for the NASH model). Before PA treatment, the HepG2 cells were pretreated with AI (50 or 100 *μ*g/mL), CL (50 or 100 *μ*g/mL), ACE (25 or 50 *μ*g/mL), or positive control (curcumin 5 *μ*g/mL or scopoletin 5 *μ*g/mL) for 4 h. The concentrations of ACE, AI, CL, scopoletin, and curcumin were based on a screening test using a cytotoxicity assay (Supplementary Figure 1 in Supplementary Material available online at https://doi.org/10.1155/2017/4390636).

### 2.5. Oil Red O Staining in HepG2 Cells

For Oil Red O staining, cells were cultured in a 24-well plate (5 × 10^4^ per well) for 24 h. The cells were treated with 200 *μ*M PA for 24 h, after pretreatment with AI (100 *μ*g/mL), CL (100 *μ*g/mL), ACE (25 or 50 *μ*g/mL), curcumin (5 *μ*g/mL), or scopoletin (5 *μ*g/mL) for 4 h. All cells were fixed with 10% formalin and incubated for at least 1 h at room temperature. After washing the fixed cells, 60% isopropanol was added for 5 min at room temperature. After washing, cells were stained with Oil Red O working solution for 30 min and examined under an optical microscope (×100 magnifications).

### 2.6. Determination of Triglyceride and Total Cholesterol in HepG2 Cells

Intracellular triglyceride (TG) levels and total cholesterol (TC) were measured according to an enzymatic determination method as described in a previous study [20]. Cells were cultured in 100 mm culture dishes (1 × 10^6^) for 24 h. The cells were treated with 200 *μ*M PA for 24 h after pretreatment with AI (50 or 100 *μ*g/mL), CL (50 or 100 *μ*g/mL), ACE (25 or 50 *μ*g/mL), curcumin (5 *μ*g/mL), or scopoletin (5 *μ*g/mL) for 4 h. All of cells were mixed in 10% Triton X-100, and TG and TC levels were determined using commercial kits (ASAN, Korea).

### 2.7. Cell Proliferation Assay in HepG2 Cells

Cell proliferation was measured with the CCK-8 kit using the WST-8 reagent according to the manufacturer's protocol (Dojindo, Kumamoto, Japan). Briefly, cells were cultured in a 96-well plate (2 × 10^3^ per well) for 24 h. After pretreatment with AI (50 or 100 *μ*g/mL), CL (50 or 100 *μ*g/mL), ACE (25 or 50 *μ*g/mL), curcumin (5 *μ*g/mL), or scopoletin (5 *μ*g/mL) for 4 h, the cells were treated with 300 *μ*M PA for 24 h. For measurement of cell viability, 10% WST-8 reagent was added and incubated for 90 min, and absorbance at 450–600 nm the supernatants (150 *μ*L total volume) was then measured using a Soft Max 5.1 plate Reader (Molecular Devices, Sunnyvale, CA, USA).

### 2.8. Apoptosis Analysis Using Flow Cytometry in HepG2 Cells

Cells were cultured in 100 mm culture dishes (1 × 10^6^) for 24 h. The cells were treated with 300 *μ*M PA for 6 h after being pretreated with AI (100 *μ*g/mL), CL (100 *μ*g/mL), ACE (25 or 50 *μ*g/mL), curcumin (5 *μ*g/mL), or scopoletin (5 *μ*g/mL) for 4 h. All cells were harvested and washed twice with 0.1% FBS in ice-cold PBS. The cells were centrifuged (900 rpm for 5 min), then fixed in 5 mL cold 70% ethanol, and stored at 4°C until analysis. On the day of the analysis, the fixed cells were washed twice and resuspended in 1 mL FBS containing 0.1% PBS. A cell cycle analysis was conducted using the FACS system (BD Biosciences, San Jose, CA, USA) after incubation with RNase (100 *μ*g/mL) for 15 min and staining with propidium iodide (PI, 50 *μ*g/mL) at room temperature in the dark for at least 5 min. Histograms were generated, and the cell cycle analysis was carried out using Wind MDI 2.8 software (Joe Trotter, Scripps Research Institute, La Jolla, CA, USA).

### 2.9. Real-Time PCR for Gene Expression of Lipid Metabolism-Related Molecules in HepG2 Cells

Cells were cultured in 100 mm culture dishes (1 × 10^6^) for 24 h. After being pretreated with AI (100 *μ*g/mL), CL (100 *μ*g/mL), ACE (25 or 50 *μ*g/mL), curcumin (5 *μ*g/mL), or scopoletin (5 *μ*g/mL) for 4 h, the cells were treated with 200 *μ*M PA for 6 h. Total RNA from cells was extracted using the QIAzol reagent (Germantown, MD). Complementary DNA (cDNA) synthesis from the RNA was performed using a High-Capacity cDNA Reverse Transcription Kit (Ambion, Austin, TX, USA). Real-time PCR was performed on 4 genes including *β*-actin using an iQ5 instrument (Bio-Rad, USA). The primer sequences were as follows: CD36: 5′-GCC AAG CTA TTG CGA CAT GAT-3′ and 3′-GAA AAG AAT CTC AAT GTC CGA GAG T-5′, SREBP1c: 5′-GAG CGA GCG TTG AAC TGT AT-3′ and 3′-ATG CTG GAG CTG ACA GAG AA-5′, PPAR-*γ*: 5′-AGG TGG AGA TGC AGG TTC TA-3′ and 3′-TGG GAG ATT CTC CTG TTG AC-5′ and PPAR-*α*: 5′-TGG CAA AAG GCA AGG AGA AG-3′ and 3′-CCC TCT ACA TAG AAC TGC AAG GTT T-5′.

### 2.10. Western Blot Analysis for Apoptosis-Related Molecules in HepG2 Cells

Western blot detection of 5 proteins, including GRP78, peIF2*α*, eIF2*α*, CHOP, and *β*-actin as a reference protein, was conducted, and all primary antibodies were purchased from Abcam, CA, USA. Cells were cultured in 100 mm culture dishes (1 × 10^6^) for 24 h. The cells were treated with 300 *μ*M PA for 6 h, after being pretreated with AI (100 *μ*g/mL), CL (100 *μ*g/mL), ACE (25 or 50 *μ*g/mL), curcumin (5 *μ*g/mL), or scopoletin (5 *μ*g/mL) for 4 h. The cells were lysed in RIPA buffer. Protein concentration was estimated using the Bio-Rad protein assay reagent (Hercules, CA, USA), and aliquots of cell lysates corresponding to 10 *μ*g total protein were separated by 10% SDS-polyacrylamide gel electrophoresis and blotted onto a PVDF membrane. The membranes were blocked with 5% skim milk for 40 min and incubated with primary antibodies overnight and then washed with 0.1% PBST. The membranes were then incubated for 1 h with secondary antibody conjugated with peroxidase and washed with PBST. The signals were detected with a visually enhanced detector using a chemiluminescent detection system (MY ECL Imager, Thermo Scientific Co., San Jose, CA, USA).

### 2.11. Statistical Analysis

Data are expressed as the means ± SD. Differences between groups were assessed using one-way analysis of variance and Fisher's least-significant difference test. In all analyses, *P* < 0.05 or *P* < 0.01 was used as a threshold to indicate statistical significance.

## 3. Results

### 3.1. Compositional Analysis of ACE, AI, and CL

The compositional analyses for the main chemicals components of ACE, AI, and CL were performed using UHPLC-MS/MS. The histogram of ACE indicated that four types of flavonoid family chemicals were detected, including scopoletin, bisdemethoxycurcumin, demethoxycurcumin, and curcumin at 9.04, 22.72, 23.38, and 24.04 min of retention time, respectively. Quantitative analysis of the above chemicals was conducted using a UHPLC-MS/MS system, and their quantities were as follows: 0.635 (scopoletin), 0.039 (bisdemethoxycurcumin), 0.016 (demethoxycurcumin), and 0.037 (curcumin) *μ*g/mg. ACE contained all the chemical components of both AI and CL (Figure 1 and Table 1).

### 3.2. Histological Analysis of Fat Accumulation

**Figure 2:**
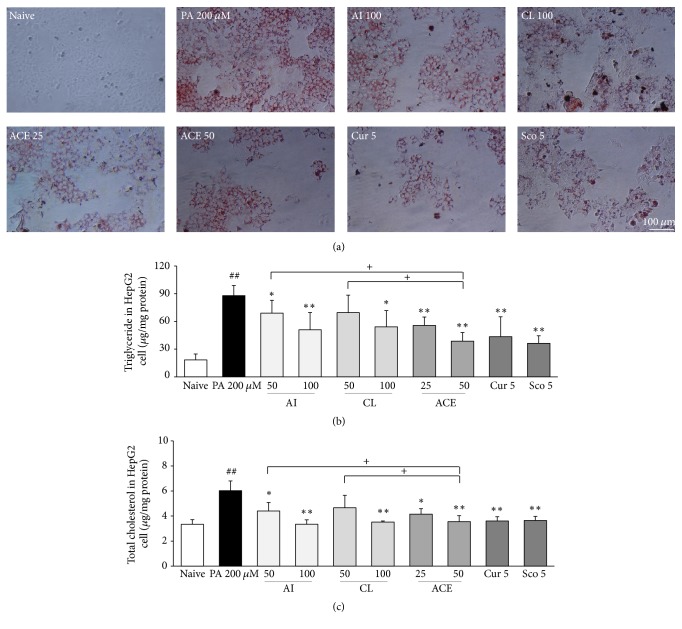
Oil Red O staining and lipid profiles in HepG2 cells. (a) HepG2 cells evaluated by Oil Red O staining. All images were obtained under 200x magnification. (b) Triglyceride and (c) total cholesterol were determined. The data are expressed as the mean ± SD (*n* = 6). ^##^*P* < 0.01 compared with nontreatment cells; ^*∗*^*P* < 0.05, ^*∗∗*^*P* < 0.01 compared with PA-treated cells; ^+^*P* < 0.05 compared with ACE 50.

PA treatment (200 *μ*M for 24 h) drastically increased fat accumulation in HepG2 cells compared with nontreated cells based on Oil Red O staining. The pretreatment with ACE (25 or 50 *μ*g/mL) notably attenuated fat accumulation compared with PA treatment-only cells. This beneficial pattern was also observed in treatment with 100 *μ*g/mL of AI or CL or with 5 *μ*g/mL of curcumin or scopoletin (Figure 2(a)).

### 3.3. Effects on Total Cholesterol and Triglyceride Levels

PA treatment (200 *μ*M for 24 h) dramatically elevated the quantity of TC (1.8-fold) and TG (4.9-fold) in hepG2 cells compared with the nontreated cells. Pretreatment with ACE significantly ameliorated the intracellular accumulation of both TG (*P* < 0.01 for both 25 *μ*g/mL and 50 *μ*g/mL) and TC (*P* < 0.05 for 25 *μ*g/mL and *P* < 0.01 50 *μ*g/mL). These beneficial results were also observed in pretreatment with AI, CL, curcumin, or scopoletin, but ACE (50 *μ*g/mL) was significantly superior to 50 *μ*g/mL AI or CL (*P* < 0.05 for TG and TC, Figures 2(b) and 2(c)).

### 3.4. Effects on Cell Proliferation

**Figure 3:**
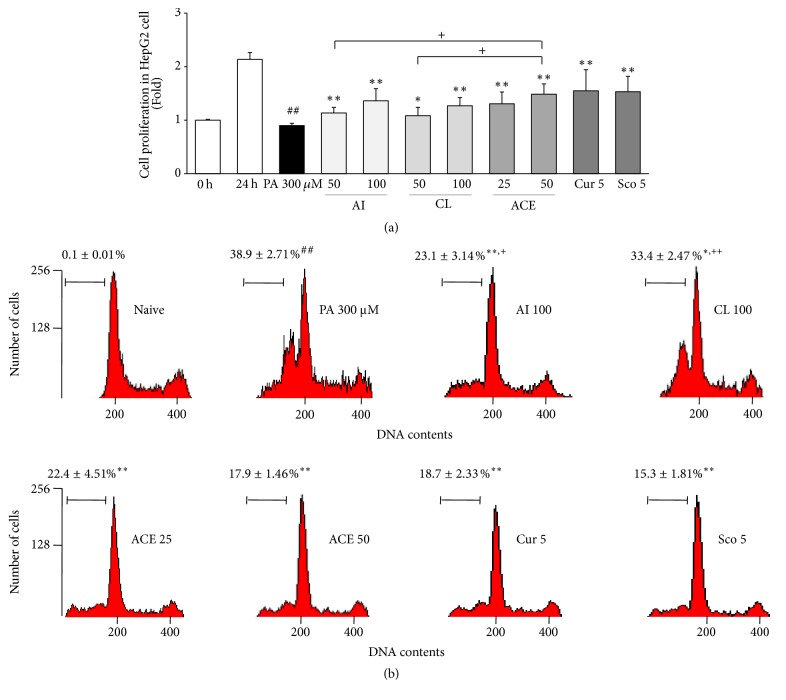
Cytotoxicity assay in HepG2 cells. (a) Cell proliferation and (b) cell cycle arrest assay were determined in HepG2 cells. The data are expressed as the mean ± SD (*n* = 6). ^##^*P* < 0.01 compared with nontreatment cells; ^*∗*^*P* < 0.05, ^*∗∗*^*P* < 0.01 compared with PA-treated cells; ^+^*P* < 0.05, ^++^*P* < 0.01 compared with ACE 50.

PA treatment (300 *μ*M for 24 h) completely inhibited proliferation of HepG2 cells. This lipotoxicity was significantly attenuated by pretreatments with ACE (*P* < 0.01 for 25 *μ*g/mL and 50 *μ*g/mL). The pretreatments with AI, CL, curcumin, or scopoletin also showed those protective effects, and then ACE (50 *μ*g/mL) was significantly superior to 50 *μ*g/mL of AI or CL (*P* < 0.05, Figure 3(a)).

### 3.5. Effects on Cell Apoptosis

PA treatment (300 *μ*M for 6 h) drastically increased the apoptotic cell population by approximately 39% compared with the nontreated cells. The number of apoptotic cells was significantly attenuated by pretreatments with ACE (*P* < 0.01 for 25 *μ*g/mL and 50 *μ*g/mL). AI, CL, curcumin, or scopoletin also had significant effects against PA-induced apoptosis, but ACE (50 *μ*g/mL) was significantly superior to 100 *μ*g/mL AI or CL (*P* < 0.05, Figure 3(b)).

### 3.6. Effects on Gene Expressions Related to Lipid Metabolism

**Figure 4:**
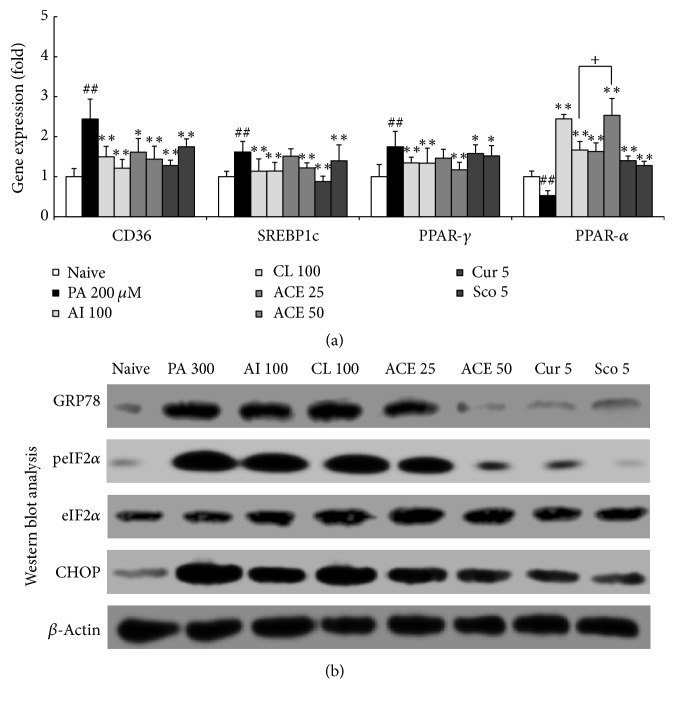
Gene expression and western blotting analyses of HepG2 cells. (a) mRNA expression levels of CD36, SREBP-1c, PPAR-*γ*, and PPAR-*α* were determined by quantitative real-time PCR. (b) Relative protein levels of GRP78, peIF2*α*, eIF2*α*, and CHOP were determined using western blotting. Data are expressed as the means ± SD (fold change relative to naïve group). ^##^*P* < 0.01 compared with nontreatment cells; ^*∗*^*P* < 0.05, ^*∗∗*^*P* < 0.01 compared with PA-treated cells; ^+^*P* < 0.05 compared with ACE 50.

The PA treatment (200 *μ*M for 6 h) markedly upregulated CD36 (2.5-fold), SREBP1c (1.8-fold), and PPAR-*γ* (1.9-fold) but downregulated PPAR-*α* (0.6-fold) compared with nontreated cells. The altered expression of these genes was significantly attenuated by the pretreatment with ACE (*P* < 0.05 or 0.01 for CD36, SREBP1c, PPAR-*γ*, and PPAR-*α*, Figure 4(a)). AI, CL, curcumin, or scopoletin also showed significant effects similar to ACE, but 50 *μ*g/mL ACE was significantly superior to 100 *μ*g/mL CL or the positive control compounds (curcumin and scopoletin).

### 3.7. Effects on Proapoptotic Proteins

The PA treatment (300 *μ*M for 6 h) notably activated the endoplasmic reticulum (ER) stress-related proapoptotic proteins, including GRP78, peIF2*α*, and CHOP, in HepG2 cells compared with nontreated cells. Pretreatment with ACE (especially 50 *μ*g/mL) considerably reduced the activation of those proteins. This pattern was observed in cells pretreated with curcumin or scopoletin, but not with AI or CL alone (Figure 4(b)).

## 4. Discussion

We adapted palmitic acid (PA) as an inducer of NAFLD and NASH models. PA is the major component of plasma free fatty acids (FFA), and serum concentration of PA is approximately 100 *μ*M in healthy subjects and approximately 200 *μ*M in obese patients [21]. To design an intracellular fat accumulation and lipotoxicity model, the present study used 200 and 300 *μ*M PA. PA enters HepG2 cells through its receptor (CD36), and excessive influx of PA both activates lipogenesis molecules such as SREBP1c and PPAR-*γ* and inactivates lipolysis molecules such as PPAR-*α* [22, 23]. As expected, treatment with PA (200 *μ*M) resulted in the upregulation of the gene expression levels of CD36, SREBP1c, and PPAR-*γ* but the downregulation of PPAR-*α* (Figure 4(a)). These results were consistent with the dramatic accumulations of intracellular triglyceride (TG), total cholesterol (TC), and the degree of Oil Red O staining. Pretreatment with ACE significantly attenuated the gene expression alterations, as well as the intracellular levels of TC and TG (Figures 2 and 4(a)). These results are consistent with previous animal studies of hyperlipidemia and arteriosclerosis models [12, 24].

Overaccumulation of fat droplets is known to generate oxidative stressors such as reactive oxygen species and nitric oxide and to induce the inflammatory response in hepatic tissue [25]. In addition, endoplasmic reticulum (ER) stress and mitochondrial dysfunction ultimately lead to apoptosis of hepatic cells, which is known as NASH [26]. As shown in our results, treatment with 300 *μ*M PA caused prominent apoptotic death of HepG2 cells, as had already been reported by others [27]. As expected, we found that ACE had statistically significant anti-NASH effects (Figure 3).

In addition to effects on key molecules in lipid metabolism, we further examined three molecules related to ER stress to explore the pharmaceutical action of ACE. The hepatic steatosis and ER stress have a bad influence on each other in pathological process, and ER stress is known to be a main mechanism in NASH [28]. Approximately 10% of subjects with NAFLD eventually develop of NASH [2]. Glucose-regulated protein 78 (GRP78) regulates several initiators of ER stress such as protein kinase RNA-like endoplasmic reticulum kinase (PERK), inositol requiring enzyme 1*α* (IRE1*α*), and eukaryotic initiation factor 2 (eIF2) [29, 30]. These molecules mediate the apoptosis of hepatocytes via the CCAAT-enhancer-binding protein homologous protein (CHOP) pathway in NASH [31]. Our results showed that treatment with PA increased protein levels of GRP78, eIF2*α*, and CHOP, whereas pretreatment with ACE attenuated these alterations (Figure 4(b)).

From UHPLC-MS/MS analysis, we confirmed that the main compounds of ACE, AI, and CL were scopoletin, bisdemethoxycurcumin, demethoxycurcumin, and curcumin, respectively (Figure 1 and Table 1). We used scopoletin and curcumin as the positive controls, because these compounds have been reported to inactivate lipogenesis and to activate lipolysis [32, 33]. Accordingly, an anti-NAFLD or anti-NASH effect was expected. We consistently found the synergistic efficacy of ACE was greater compared with AI or CL alone. Regarding intracellular fat accumulation, 50 *μ*g/mL ACE had significantly higher activity compared with the same dose (50 *μ*g/mL) of AI or CL alone. Regarding hepatocyte apoptosis, 50 *μ*g/mL ACE was far superior to AI or CL (100 *μ*g/mL) as well as scopoletin and curcumin (5 *μ*g/mL). The quantities of scopoletin and curcumin in 50 *μ*g of ACE were approximately 31 ng and 1.9 ng, respectively. We can suppose that ACE contains other active compounds besides scopoletin and curcumin. In addition, the synergic action resulted from the combination of AI and CL, which are the representative herbs for treating* “dampness and phlegm”* (濕痰) and* “blood stasis”* (瘀血) in KTM, respectively.

We conclude that the combination of* A. iwayomogi* and* C. longa* radix has a synergic effect on NAFLD and NASH, and its corresponding mechanisms involve the regulations of lipid metabolism and ER stress molecules. Further studies are needed to identify the active compounds and the details of the molecular pathways mediating these responses.

## Supplementary Material

HepG2 Cells (2 × 10^3^) were seeded to 96-well plates with DMEM. Then, cells were treated with ACE, AI, CL, Sco or Cur 24 h at concentrations given. Cell proliferation was determined in HepG2 cells using a WST assay.

## Figures and Tables

**Table 1:** Retention time and contents of sample in ACE, AI, and CL.

Number	Compound	Retention time (min)	ACE		AI		CL	
			Mean	SD	Mean	SD	Mean	SD
			(*µ*g/mg)		(*µ*g/mg)		(*µ*g/mg)	
(1)	Scopoletin	9.04	0.635	0.0047	0.730	0.0043	ND	ND
(2)	Bisdemethoxycurcumin	22.72	0.039	0.0026	ND	ND	0.047	0.0031
(3)	Demethoxycurcumin	23.38	0.016	0.0026	ND	ND	0.032	0.0021
(4)	Curcumin	24.04	0.037	0.0034	ND	ND	0.070	0.0028

## Abbreviations

ACE: Combined extract of* Artemisia iwayomogi* and* Curcuma longa*
AI: * Artemisia iwayomogi* extract
CHOP: CCAAT-enhancer-binding protein homologous protein
CL: * Curcuma longa* extract
eIF2: Eukaryotic initiation factor 2
ER: Endoplasmic reticulum
GRP78: Glucose-regulated protein 78
IRE1*α*: Inositol requiring enzyme 1*α*
NAFLD: Nonalcoholic fatty liver diseases
NASH: Nonalcoholic steatohepatitis
PA: Palmitic acid
PERK: Protein kinase RNA-like endoplasmic reticulum kinase
PPAR-*α*: Peroxisome proliferator-activated receptor alpha
PPAR-*γ*: Peroxisome proliferator-activated receptor gamma
SREBP1c: Sterol regulatory element-binding protein factor 1 type c
TC: Total cholesterol
TG: Triglyceride
TKM: Traditional Korean medicine
UHPLC-MS/MS: Ultra-high-performance liquid chromatography-tandem mass spectrometry.
