# Supplementary material for: Artemisia iwayomogi plus Curcuma longa Synergistically Ameliorates Nonalcoholic Steatohepatitis in HepG2 Cells
Source: Evid Based Complement Alternat Med. 2017 Oct 17;2017:4390636. doi: 10.1155/2017/4390636 (PMC5664258; doi:10.1155/2017/4390636)
Supplement: Supplementary file 1 — HepG2 Cells (2 × 10^3^) were seeded to 96-well plates with DMEM. Then, cells were treated with ACE, AI, CL, Sco or Cur 24 h at concentrations given. Cell proliferation was determined in HepG2 cells using a WST assay. [file 4390636.f1.docx]

**Supplementary material.**

HepG2 Cells (2 × 10^3^) were seeded to 96-well plates with DMEM. Then, cells were treated with ACE, AI, CL, Sco or Cur 24 h at concentrations given. Cell proliferation was determined in HepG2 cells using a WST assay.

**Supplementary Figure**


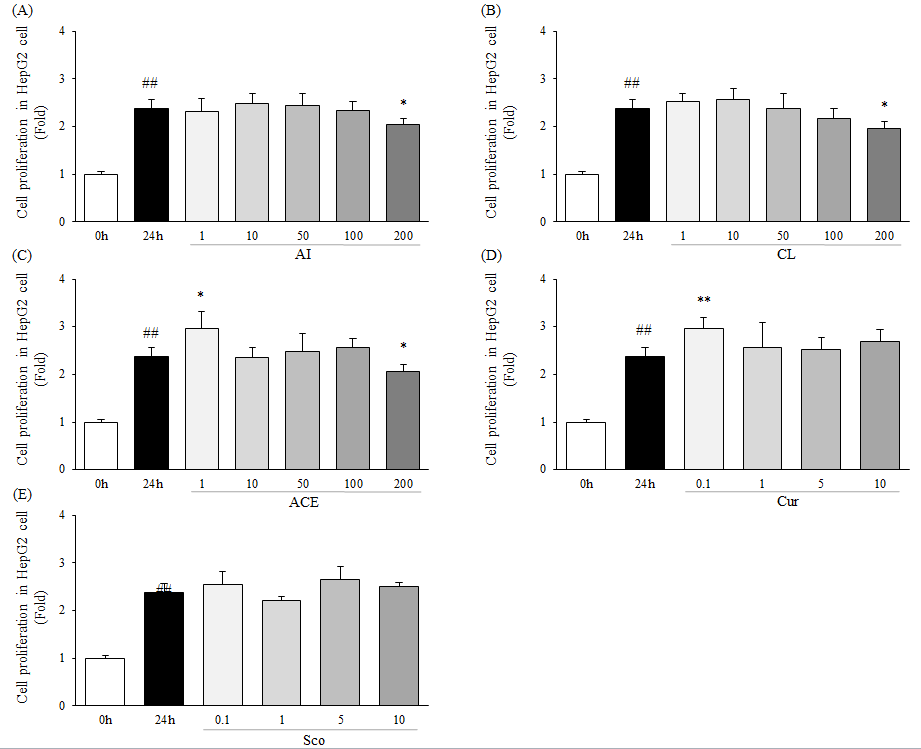


**Supplementary Figure 1. Cytotoxicity of ACE, AI, CL, Sco and Cur in HepG2 cells.** Cell proliferation was determined in HepG2 cells using a WST assay. HepG2 Cells (2 × 10^3^) were seeded to 96-well plates with DMEM. Then, cells were treated with ACE, AI, CL, Sco or Cur 24 h at concentrations given. The data are expressed as the mean ± SD (*n=6*). ^##^*p* < 0.01, compared with the 0 h group; ^*^*p* < 0.05, ^**^*p* < 0.01, compared with the non-treatment group. ACE; *Artemisia iwayomogi* plus *Curcuma longa*, AI*; Artemisia iwayomogi*, CL*; Curcuma longa*, Cur; Curcumin, Sco; scopolectin
